# Supplementary material for: Causal Relationship between Aging and Anorexia Nervosa: A White-Matter-Microstructure-Mediated Mendelian Randomization Analysis
Source: Biomedicines. 2024 Aug 16;12(8):1874. doi: 10.3390/biomedicines12081874 (PMC11351342; doi:10.3390/biomedicines12081874)
Supplement: Supplementary file 1 [file biomedicines-12-01874-s001.zip › Supplementary Figure/Figure S4/Singe SNP plot of MR analysis for TL to ubm-b-1630.pdf]

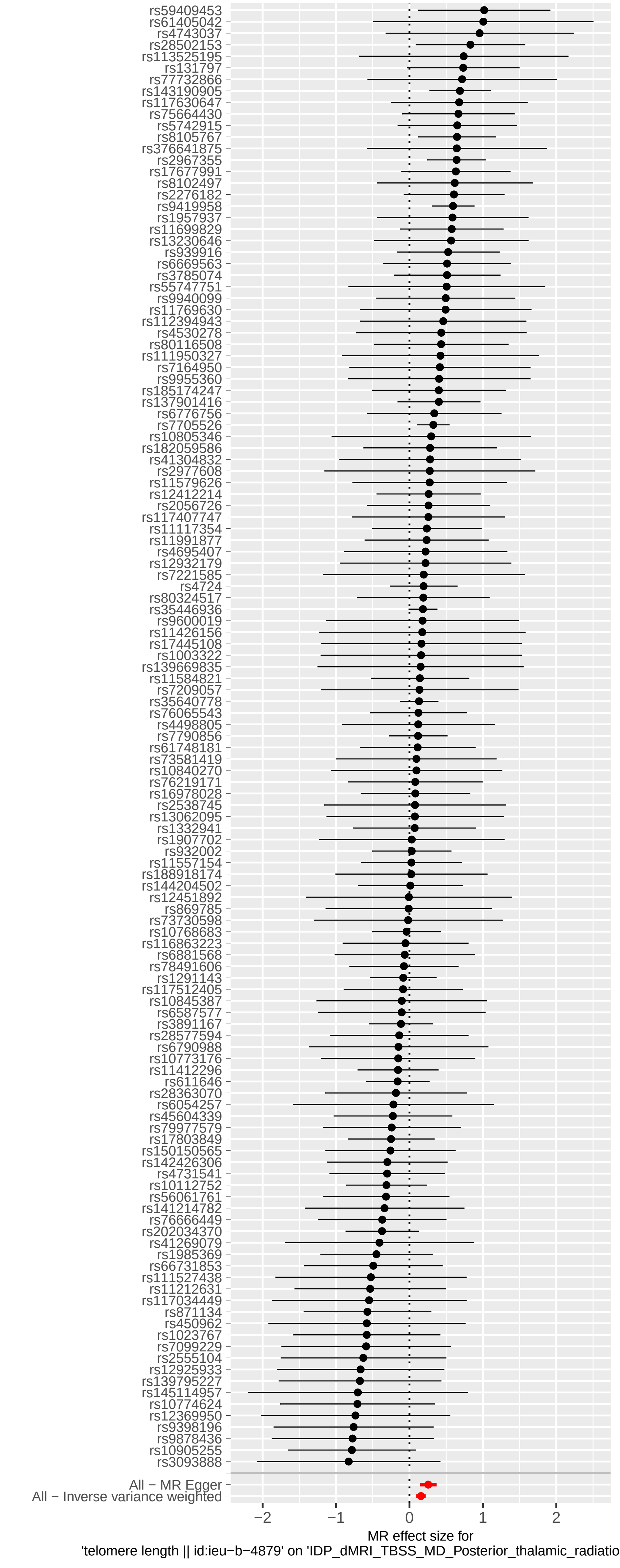
rs59409453
rs61405042
rs4743037
rs28502153
rs113525195
rs131797
rs77732866
rs143190905
rs117630647
rs75664430
rs5742915
rs8105767
rs376641875
rs2967355
rs17677991
rs8102497
rs2276182
rs9419958
rs1957937
rs11699829
rs13230646
rs939916
rs6669563
rs3785074
rs55747751
rs9940099
rs11769630
rs112394943
rs4530278
rs80116508
rs111950327
rs7164950
rs9955360
rs185174247
rs137901416
rs6776756
rs7705526
rs10805346
rs182059586
rs41304832
rs2977608
rs11579626
rs12412214
rs2056726
rs117407747
rs11117354
rs11991877
rs4695407
rs12932179
rs7221585
rs4724
rs80324517
rs35446936
rs9600019
rs11426156
rs17445108
rs1003322
rs139669835
rs11584821
rs7209057
rs35640778
rs76065543
rs4498805
rs7790856
rs61748181
rs73581419
rs10840270
rs76219171
rs16978028
rs2538745
rs13062095
rs1332941
rs1907702
rs932002
rs11557154
rs188918174
rs144204502
rs12451892
rs869785
rs73730598
rs10768683
rs116863223
rs6881568
rs78491606
rs1291143
rs117512405
rs10845387
rs6587577
rs3891167
rs28577594
rs6790988
rs10773176
rs11412296
rs611646
rs28363070
rs6054257
rs45604339
rs79977579
rs17803849
rs150150565
rs142426306
rs4731541
rs10112752
rs56061761
rs141214782
rs76666449
rs202034370
rs41269079
rs1985369
rs66731853
rs111527438
rs11212631
rs117034449
rs871134
rs450962
rs1023767
rs7099229
rs2555104
rs12925933
rs139795227
rs145114957
rs10774624
rs12369950
rs9398196
rs9878436
rs10905255
rs3093888
All − MR Egger
All − Inverse variance weighted
−2
−1
0
1
2
MR effect size for
'telomere length || id:ieu−b−4879' on 'IDP_dMRI_TBSS_MD_Posterior_thalamic_radiatio
